# Technological Solutions to Address Drug-Resistant *Neisseria gonorrhoeae*

**DOI:** 10.3201/eid2205.160083

**Published:** 2016-05

**Authors:** Claire C. Bristow, Huan Dong, Jeffrey D. Klausner

**Affiliations:** University of California San Diego, La Jolla, California, USA (C.C. Bristow);; Charles R. Drew University of Medicine and Science, Los Angeles, California, USA (H. Dong);; University of California Los Angeles, Los Angeles (H. Dong, J.D. Klausner)

**Keywords:** Neisseria gonorrhoeae, gonorrhoea, gonorrhea, antimicrobial resistance, antibiotic resistance rates, bacteria, cephalosporins, susceptibility, resistance, azithromycin, sexually transmitted infections, STD, Canada

**To the Editor:** Since the 1930s, *Neisseria gonorrhoeae* has become resistant to drugs in every class of antimicrobial therapy used to treat it. We read with interest the article by Martin et al. about trends in Canada on *N*. *gonorrhoeae* susceptibility to third-generation cephalosporins, the only class of antimicrobial drugs to which most *N*. *gonorrhoeae* strains remain susceptible (*1*). We find the reported decrease in cefixime- and ceftriaxone-reduced susceptibility during 2010–2014 encouraging, but remain concerned about a threat from drug-resistant and untreatable *N*. *gonorrhoeae* infections: a similar downward trend in the United States reversed in 2014 (*2*). That divergence demonstrates the limited reliability of surveillance data.

Addressing resistance requires new methods for susceptibility determination without culture. Real-time screening for genes associated with antimicrobial drug resistance, such as *penA* mosaic alleles yielding decreased susceptibility to oral extended-spectrum cephalosporins, may be a valuable method to determine treatment (*3*). In the same issue of Emerging Infectious Diseases, Deguchi et al. described a case of multidrug-resistant *N*. *gonorrhoeae* (*4*), further highlighting the urgency for the innovative approach of using molecular tests to individualize treatment regimens. An ongoing study supported by the National Institutes of Health (R21AI109005) is evaluating how a laboratory-developed molecular *N*. *gonorrhoeae* genotypic susceptibility test for ciprofloxacin enables rapid identification of effective antimicrobial drugs (*5*).

*N*. *gonorrhoeae* may acquire new resistance mechanisms under selection pressures imposed by use of antimicrobial drugs and horizontal gene transfer from other commensal *Neisseria* species resident in the human oropharynx (*3*). Inconsistent pharyngeal *N*. *gonorrhoeae* screening may lead to missed opportunities for treatment. A National Institutes of Health program (Antibiotic Resistance Leadership Group, award no. UM1AI104681) is ongoing to assist manufacturers in obtaining US Food and Drug Administration approval for molecular assays to detect extragenital gonococcal infections.

For nearly 8 decades, *N. gonorrhoeae* has been controllable. Continued investment in research and the development of new laboratory technology are critical in supporting an effective response to mitigate the threat of untreatable gonorrhea.
